# Analysis of the structural continuity in twinned crystals in terms of pseudo-eigensymmetry of crystallographic orbits

**DOI:** 10.1107/S2052252513026493

**Published:** 2013-10-18

**Authors:** Mohamed Amine Marzouki, Bernd Souvignier, Massimo Nespolo

**Affiliations:** a Radboud University Nijmegen, Faculty of Science, Mathematics and Computing Science, Institute for Mathematics, Astrophysics and Particle Physics, Postbus 9010, 6500 GL Nijmegen, The Netherlands; b Université de Lorraine, Faculté des Sciences et Technologies, Institut Jean Barriol FR 2843, CRM2 UMR CNRS 7036, BP 70239, Boulevard des Aiguillettes, F-54506 Vandoeuvre-lès-Nancy cedex, France

**Keywords:** melilite, structural continuity, twinned crystals

## Abstract

The reticular theory of twinning gives the necessary conditions on the lattice level for the formation of twins. The latter are based on the continuation, more or less approximate, of a substructure through the composition surface. The analysis of this structural continuity can be performed in terms of the eigensymmetry of the crystallographic orbits corresponding to occupied Wyckoff positions in the structure. If  is the space group of the individual and  a space group which fixes the twin lattice obtained as an intersection of the space groups of the individuals in their respective orientations, then a structural continuity is obtained if (1) the eigensymmetry of an orbit under  contains the twin operation; (2) the eigensymmetry of a union of orbits under  contains the twin operation; (3) the eigensymmetry of a split orbit under  contains the twin operation; or (4) the eigensymmetry of a union of split orbits under  contains the twin operation. The case of the twins in melilite is analysed: the (approximate) restoration of some of the orbits explains the formation of these twins.

## Abstract 2

A general approach to the analysis of structural continuity in twins is presented and applied to the known twins in melilite.

## 1. Symbols   

(**a**, **b**, **c**): basis vectors of the unit cell.

*a*, *b*, *c*: length of basis vectors.

*r_i_* = 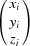: coordinates of the *i*th crystallographically independent atom *A_i_*.


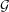
: space group of the individual, 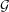 = {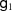, 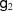, …}, with 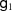 = 1 the identity element of 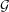.

*O_i_*: orbit of *r_i_* under 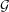, *O_i_* = {*r_i_*, 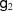 *r_i_*, …} = {*r_i_* ^1^, *r_i_* ^2^, …} with *r_i_^k^* = 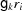 for 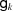 ∈ 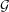.

*O_ij_*: splitting of the orbit *O_i_* under the action of a subgroup of 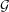.

*m*(*O_i_*): multiplicity of the orbit *O_i_*, defined as the number of equivalent points in the conventional unit cell of 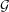.

(**P**, **p**): matrix-column pair representing a change of basis; composed of a 3 × 3 matrix **P** and a 3 × 1 column **p**.


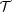
: matrix representation of the twin operation in the basis of the twin.


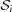
: site-symmetry group of *r_i_*.


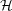
: space group associated with the structure of the twin.


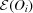
: eigensymmetry of the orbit *O_i_*.

## 2. Introduction   

A twin is a heterogeneous crystalline edifice composed of two or more homogeneous crystals of the same phase with different orientation related by a twin operation, *i.e.* a crystallographic operation mapping the orientation of one individual onto that of the other(s) (Friedel, 1904 ▶, 1926 ▶, 1933 ▶). A twin element is the geometric element in direct space (plane, line, centre) about which the twin operation is performed.

Twins can be classified from the genetic viewpoint in three categories:

(1) Transformation twins, which form during a phase transition leading to a loss of point symmetry.

(2) Mechanical twins, which form as the result of a mechanical action (typically, an oriented pressure) on the crystal.

(3) Growth twins, which form during crystal growth, either at the nucleation stage or by oriented attachment (for a review, see Nespolo & Ferraris, 2004*a* ▶).

For cases (1) and (2), the cause of the formation of the twin is known. For the growth twins the formation can be a response to a mistake in the normal crystal growth of the individual or the random association of two or more crystals with different orientation (non-equivalent under the symmetry group of the crystal). This category of twins appears not only during the formation of a natural crystal but also during the synthesis of artificial crystals.

The interface that separates the individuals represents a discontinuity for at least a sub-structure. This heterogeneity gives rise to serious problems in the structural study of materials and biomaterials and it represents an obstacle for structural investigations as well as for crystal engineering and material design. For example:

(*a*) The potential technological applications are hindered by the presence of twinning (*e.g*. the piezoelectric effect is reduced or annihilated).

(*b*) The presence of twinning reduces the amount of details that can be obtained from a structural study, especially for samples with large unit cells (for example, macromolecules) for which the resolution that can be achieved is already limited by the size of the unit cell.

From the viewpoint of the material scientist and of the crystal grower, the development of a synthesis protocol capable of reducing, if not suppressing, the formation of twins is an important goal. To reach this aim a detailed understanding of the formation mechanism of twins is of paramount importance.

A prerequisite for the formation of a twin is a partial structural continuity through the interface. In fact, without any structural continuity the edifice built by the individual crystals would be unstable or simply not form at all; a complete structural continuity is the feature of a single crystal; in a twin a part of the structure has to continue, more or less unperturbed, across the interface. This atomic continuity implies the continuity of a sub-lattice. In fact, the lattice represents the periodicity of the crystal pattern and the continuity of a sub-lattice is a necessary condition for the continuity of a sub-structure. The reticular approach abstracts from the structure and estimates the lattice restoration by the twin operation in terms of the twin index and the obliquity. A good restoration of the lattice is a necessary but not sufficient condition to obtain a good structure restoration. The latter would enhance the reticular theory to conditions which are *structurally necessary* for the formation of twinned crystals. A general theory on this has not been developed yet.

Extensive research from the lattice viewpoint during more than a century led to the reticular theory developed by Bravais (1851 ▶), Mallard (1885 ▶) and Friedel (1904 ▶, 1926 ▶), based on the existence of a common (sub)-lattice in the three dimensions of the crystallographic point space (note however the special case of monoperiodic twins reported by Friedel, 1933 ▶). The common (sub)-lattice, called the twin lattice (Donnay, 1940 ▶), is based on the twin element (twin plane or twin axis) and the lattice element (line or plane) that are mutally (quasi)-perpendicular. The twin lattice **L** *_T_* is defined by these two elements (*hkl*)*_T_* and [*uvw*]*_T_*. When the two elements are reciprocally perpendicular one speaks of twin lattice symmetry (TLS: Donnay & Donnay, 1974 ▶) and the two elements are symmetry elements for **L** *_T_*. Otherwise one speaks of twin lattice quasi symmetry (TLQS: Donnay & Donnay, 1974 ▶); the two elements are only pseudo-symmetry elements for **L** *_T_*. The degree of pseudo-symmetry corresponds to the deviation from the perpendicularity condition and is measured by the angle ω called the *obliquity*.^1^ The twin index *n* is the inverse of the fraction of lattice nodes restored by the twin operation and corresponds to the ratio between the volumes of the primitive cells of the twin and the individual, *n* = *V*(**L** *_T_*)/*V*(**L**_ind_). Friedel gave as empirical limits for the occurrence of twins *n* ≤ 6 and ω ≤ 6. Twins falling within these limits are called *Friedelian twins* (Nespolo & Ferraris, 2005 ▶). The frequency of occurrence of a twin depends on the degree of lattice restoration: the lower the twin index and the obliquity, the better is the lattice restoration and the higher is the probability that the twin actually occurs. This relation between the occurrence frequency of twins and the values of *n* and ω is an empirical observation, based, however, on the extensive study of twins over more than a century. It shows the necessary (not sufficient) character of the lattice restoration. Nevertheless some twins with higher index are known that violate the empirical limits: they are called non-Friedelian twins (Nespolo & Ferraris, 2005 ▶). These twins seem to contradict the general conclusion that a high degree of lattice restoration is a necessary condition for a twin to form. However, in most cases they can be explained by the fact that two or more sublattices contribute to the lattice quasi-restoration. When all the concurrent sublattices are taken into account, the necessary conditions are no longer contradicted. The interpretation of the occurrence of this kind of twins is the object of the hybrid theory of twinning (Nespolo & Ferraris, 2005 ▶), which represents an extension of the reticular theory and measures the lattice quasi-restoration in terms of an *effective twin index n*_E_ (Nespolo & Ferraris, 2006 ▶), a real number defined as the ratio between the lattice nodes of the individual and the lattice nodes belonging to any of the quasi-restored sublattices. In the case of a single quasi-restored sublattice, this coincides with the classical twin index; otherwise it is lower. In the few examples which are neither explained by the classical reticular nor by the hybrid theory of twinning, the possibility of a wrong choice of the twin element has to be considered (reflection twins in place of rotation twins or *vice versa*). This indeed resolves the apparent contradiction of a higher frequency of twins with higher index than twins with a lower index observed in some cases like the staurolite twins. The Saint Andrews cross twin of staurolite, with index *n* = 12, is more frequent than the Greek cross twin with index *n* = 6 (Nespolo & Ferraris, 2007 ▶). These twins are often reported as reflection twins on (031) and (231), respectively, but experimental studies have shown (Hurst *et al.*, 1956 ▶) that this interpretation is incorrect and that they actually are rotation twins. For the Saint Andrews cross twin (*n* = 12), the correct choice of the twin element as a line shows the existence of two lattice planes quasi-perpendicular to it and correspondingly two sublattices are quasi-restored by the twin operation. This gives an effective index *n*_E_ = 6.0 and as a consequence the Saint Andrews twin is brought back into the Friedelian limits. The occurrence frequency no longer contradicts the necessary condition of a good lattice restoration (Nespolo & Ferraris, 2009 ▶).^1^

The reticular theory of twinning can only provide partial prerequisites for the formation of twins, which are governed by the structure. More conclusive conditions can only be obtained by the analysis of the structural coherence at the interface, but such an analysis reduces to a case-by-case *a posteriori* study of known twins. Our purpose is to develop a general structural theory of twinning to predict the structurally necessary conditions for the formation of twins in a general way through an algebraic algorithm. A twin fulfilling these conditions *can* form (and may even be likely to form), but does not necessarily have to form. Indeed, a growth twin is a ‘mistake’ originated by defects or perturbation of growth conditions and does not correspond to the thermodynamically most stable situation (Buerger, 1945 ▶). Donnay & Curien (1960 ▶) were the first to suggest the application of the analysis of the eigensymmetry of crystallographic orbits, in the case of pyrite and digenite, which led to the introduction of a restoration index for a subset of atoms (Takeda *et al.*, 1967 ▶). This subset must be quasi-continuous across the interface, otherwise the interface would be incoherent, the contact between the individuals would be unstable and the twin would not form. Under the action of the space group 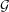, each atom in a crystal is repeated in space to form a crystallographic orbit *O*, *i.e.* *O* is the set of all atoms obtained under the symmetry operations of the space group 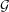. The eigensymmetry 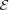
(*O*) of the orbit may be a supergroup of 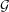 or coincide with it; accordingly, crystallographic orbits are classified in three types according to the relation between 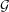 and 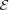:

*Characteristic orbit*: 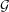 = 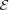.

*Non-characteristic orbit*: 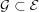 but 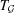 = 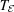.

*Extraordinary orbit*: 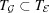, a special case of non-characteristic orbit defining a superlattice (smaller unit cell) with respect to 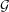.

Here 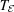 and 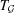 are the translation subgroups of 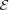 and 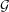, respectively. When 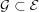, an operation *t* belonging to 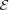 but not to 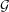 may map the orientation of crystal 1 onto that of crystal 2 and may thus serve as twin operation.

## 3. Crystallographic orbit approach to the analysis of structural continuity in twins   

Depending on the nature of the twin operation, twins can be classified into three categories:

(1) twins by reflection;

(2) twins by rotation;

(3) twins by inversion.

An inversion twin is always by (pseudo)-merohedry, *i.e*. it corresponds to twin index *n* = 1 and does not give rise to a sublattice, because the whole lattice of the individual is (quasi)-restored. For a twin with index *n* > 1, the twin operation is not about a lattice direction, which makes its matrix representation non-integral with respect to the basis of the individual. By expressing the twin operation in the basis of the twin, its representation becomes integral again.

The reticular theory of twinning shows that an exact restoration of the lattice is not an absolute condition for the twin to form, a limited departure from the restoration, measured by the obliquity or the twin misfit, being the rule rather than the exception. In the same way, we can expect that a limited departure from structural continuity at the interface does not represent a hindrance to twin formation. In the following, all the occurrences of ‘restoration’ should thus be read as ‘restoration or quasi-restoration’. As a consequence, the eigensymmetry of an orbit has to be taken with some degree of tolerance: a pseudo-eigensymmetry will result in quasi-restoration. The choice of this tolerance has clearly important consequences on the conclusions one may draw about the structural quasi-continuity. Choosing a too small tolerance may lead to a relatively good coherence at the interface being overlooked; a too large tolerance would have no real physical meaning. Clearly, the tolerance has to be chosen keeping in mind the atomic size: it is greater for a large atom than that for a small one. As a rule of the thumb, about 50% of the atomic diameter (*i.e*. the radius: ionic, covalent or atomic depending on the type of bond) seems a reasonable figure.

Let (*hkl*)*_T_* and [*uvw*]*_T_* be the mutually (quasi)-perpendicular plane and direction which define the cell of the twin lattice. Let **v**_1_ and **v**_2_ be two vectors defining a two-dimensional unit cell in (*hkl*)*_T_*. The three linearly independent vectors **v**_1_, **v**_2_ and [*uvw*]*_T_* form the twin basis, denoted by (**abc**)*_T_*, which is related to the basis (**abc**)*_I_* of the individual by the basis transformation **P**:
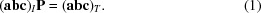



**L**_ind_ and **L** *_T_* have a common origin: there is thus no vector part in the relation between the two references. Given the coordinates (*xyz*)*_I_* of an atom in the individual basis, the new coordinates (*xyz*)*_T_* of this atom in the twin basis are obtained by the relation:
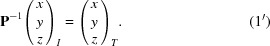



Each atom with coordinates *r_i_* generates a crystallographic orbit *O_i_* with eigensymmetry 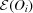 under the action of the symmetry operations of the space group 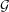. If the orbit is non-characteristic, its eigensymmetry group 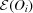 may contain the twin operation *t*, in which case the orbit is restored by the twin operation. This cannot be true for all the orbits, otherwise *t* would belong to the space group of the individual and the structure would be a single individual and not a twin. When the orbit is not fully restored, a subset of atoms belonging to the orbit can instead be restored. This subset is defined by a subgroup 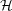 of 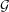 obtained by intersecting the space groups of the individuals. Since the twin index is *n* > 1, 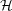 is a proper subgroup of 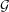, the translation subgroup of 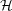 is a subgroup of index *n* in the translation subgroup of 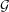.

Let 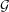 be the space group of one of the individuals of a twinned crystal. The twin operation *t* maps the first individual to the second individual (assuming, for ease of description, the case of a twofold twin) and the space group of the second individual is the conjugate group 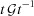. In addition, the twin operation *t* maps the lattice **L** of the first individual to the lattice *t* **L** of the other individual and the intersection 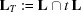 is the twin lattice. Since *t* **L** *_T_* = 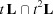 = 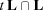 = **L** *_T_*, the twin operation fixes the twin lattice. The space group 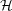 compatible with the twin lattice is the intersection of the space groups of the two individuals, written with respect to the twin basis, *i.e*. 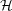 = 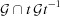. The subgroup 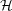 is uniquely determined; it consists of those isometries which fix both individuals separately. In particular, its translation subgroup 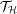 consists of the translations by vectors from the twin lattice **L** *_T_*. The above relation is easily generalized to twin operations higher than twofold by replacing **L**_1_ ∩ **L**_2_ = **L** ∩ *t* **L** with ∩*_i_* **L** *_i_* = ∩*_i_t_i_* **L**_1_.

To find the elements of 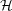, let *W* *_i_*, *w* *_i_* be the linear and translation parts of a symmetry operation of the first individual, written with respect to the twin basis, *i.e*. (*W* *_i_*, *w* *_i_*) ∈ **P** ^−1^ 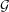 **P**. Since the linear parts of a space group act on its translation lattice, the elements belonging to 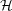 necessarily have an integral linear part *W* *_i_*. Moreover, if (*W* *_i_*, *w* *_i_*) belongs to the intersection, the conjugate (*W* *_j_*, *w* *_j_*) = 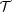
(*W* *_i_*, *w* *_i_*)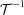 must be an element of the form (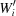, 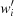) ∈ **P** ^−1^ 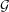 **P**. Choosing an element (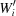, 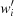) with 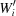 = *W* *_j_*, one finally has to check whether *w* *_j_* − 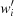 ∈ **L** *_T_*. Since the translations in 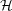 are by vectors in **L** *_T_*, two elements (*W* *_i_*, *w* *_i_*) and (*W* *_i_*, 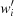) with the same linear part can only belong to 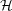 if *w* *_i_* − 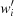 ∈ **L** *_T_*. This means that for a given element (*W* *_i_*, *w* *_i_*) of **P** ^−1^ 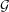 **P** one has to check elements of the form (*W* *_i_*, *w* *_i_* + *v*) for coset representatives *v* of **L** with respect to **L** *_T_*.

The study of the orbit behaviour in the twin basis is characterized by the subgroup 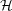 and the matrix **P**. Considering the group–subgroup related space groups 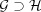, atoms which are symmetrically equivalent under 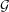, *i.e.* belong to the same orbit of 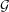, may become non equivalent under 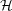 (splitting of crystallographic orbits), and/or their site-symmetry group 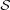 can be reduced (Wondratschek, 1993 ▶). Let *O_i_* be an orbit under 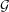, [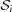, *m*(*O_i_*)] the site symmetry group and the multiplicity of the orbit with respect to the conventional cell of 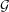, and let [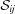, *m*(*O* *_ij_*)] be defined correspondingly for a split orbit *O_ij_* under 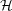, the double index indicating the original orbit under 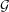 (index *i*) as well as the number of split orbits stemming from it under restriction to 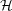 (index *j*).

In the case of splitting, the orbit *O_i_* = {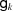 *r_i_*, 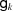 ∈ 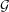} is divided into two or more orbits of 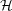, with the same/or reduced site symmetry group 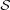 and a multiplicity equal or lower than *m*(*O_i_*). The atoms belonging to *O_i_* have 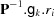 as coordinates in the twin basis. The possibilities of the splitting of the orbit *O_i_* are described by the following relations:
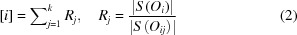
where [*i*] is the finite index of 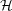 in 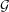, *R* *_j_* is the ratio of the order of the site-symmetry groups of the orbits *O_i_* and *O_ij_* in 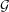 and in 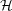, respectively, and *k* is the number of orbits in 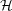 stemming from *O_i_* in 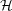 (Wondratschek, 1993 ▶).

The atomic restoration by the twin operation can finally be realised in four cases.

(1) The orbit *O_i_* is non-characteristic and its eigensymmetry 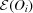 contains the twin operation *t*. In this case, **P** = **I**, where **I** is the identity matrix.

(2) The union of two or more orbits has an eigensymmetry which is higher than that of any of the orbits of the union. This may in particular happen in presence of a specialized metric corresponding, exactly or approximately, to a higher crystal family. In this case, if the twin operation is included in this higher eigensymmetry the set of atoms belonging to the union is restored although each orbit, taken separately, is not. The union can obviously be formed only from atoms with interchangable roles in the structure. For example, the union of orbits defined by crystallographically different types of oxygen, or of atoms having the same coordination environment although a different chemical species. Clearly, the fact that a different atom occurs in the same coordination on the opposite sides of the interface does not affect the structural continuity, especially if the atomic size is not extremely different. The choice of the orbits to be considered in the union must thus rely on the analysis of the structural roles of these orbits. From a formal viewpoint, the restoration occurs if *t* belongs to 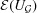 where 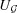 = ∪*_i_* *O_i_* and *i* spans the orbits which are not restored by *t* and are occupied by atoms with similar structural role. Here again, **P** = **I**.

(3) When neither the orbits *O_i_* nor their union 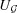 is restored, a split orbit *O_ij_* under 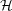 may be restored by the twin operation *t* if its eigensymmetry 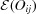 contains *t*.

(4) As in case (2) above, for orbits *O_ij_* whose 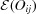 does not contain the twin operation *t*, the union 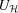 = ∪*_ij_* *O_ij_*, defined on the same criteria as 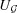, has to be considered. The restoration of a union of orbits under 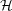 may in particular happen when the sublattice fixed by 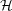 has a specialized metric corresponding, exactly or approximately, to a higher crystal family.

Cases (1) and (3) could of course be subsumed under cases (2) and (4) as unions of a single orbit or split orbit, but we emphasize the importance of these cases by discussing them separately.

The actual analysis performed is exactly the same no matter whether the group considered is 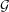 or 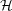 and whether we work on a single orbit or a union of orbits. Let 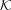 be a general notation for either 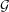 or 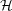 and *O* a general notation for one of *O_i_*, *O_ij_*, 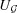 or 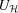. If *O* is restored by the twin operation *t*, then the eigensymmetry 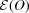 is a supergroup of 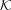 containing *t*. Such an orbit which belongs to the substructure continuing across the interface of the twin structure that is invariant under the twin operation explains (in part) the formation of the twin.

Because the eigensymmetry of (split) orbits or unions thereof is often approximate and as a consequence the restoration is imperfect, we need a quantitative measure for the degree of restoration. Let *d*_min_ be the minimal distance between the position to which a chosen atom in *O* is mapped under the twin operation *t* and the atoms in *O*. If *t* ∈ 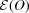, then *d*_min_ = 0 for all atoms in *O*. If *t* is only a pseudo-symmetry of *O*, then *d*_min_ > 0 and its value is a measure for the degree of quasi-restoration.

The advantage of dealing with split orbits under the intersection group 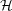 = 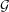 ∩ *t* 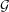 *t* ^−1^ is that the value of *d*_min_ is the same for all atoms in a split orbit under 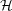, as is shown by the theorem in the Appendix A[App appa].

Let *O* ^1^ be an orbit *O* in the first individual, *O* ^2^ the corresponding orbit generated by the twin operation *t* in the second individual. The application of the twin operation *t* to *O* ^1^ generates *O* ^2^. For a fixed *orientation* of the twin element, the formation of a twin may result in a variable degree of atomic restoration depending on the *position* of the twin element in the unit cell, *i.e*. depending on which atoms are exposed to the surface or close to it. Since twinning is a point group phenomenon that occurs at a macroscopic level, the orientation of a twin element only determines the linear part of the twin operation, but not its translational part, corresponding to the position of the twin element. On the other hand, the operation which restores an orbit acts on the structure, at the microscopic (atomic) level and may well also contain an intrinsic translational part (glide or screw component). In other words, the twin operation one observes macroscopically as well as in the diffraction pattern as the overlap of differently oriented reciprocal lattices, can be realised at the atomic levels at different locations and with or without an intrinsic translation. This realisation of the twin operation is hereafter called a *restoration operation*. In order to find the possible restoration operations, one starts with the intersection group 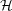 and determines its minimal supergroups which contain an operation with the required linear part. However, dealing with split orbits for the intersection subgroup 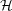 simplifies the analysis drastically. For a single split orbit and pairs of split orbits one simply checks whether the (pseudo-) eigensymmetry contains an operation of the same type as the twin operation and with its geometric element parallel to that of the twin element. The eigensymmetry analysis then provides the location of the twin element and the nature of the restoration operation.

*O* ^1^ is restored if *t* ∈ 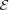
(*O* ^1^) or if *d*_min_ is lower than a certain threshold which depends on the atomic size (being smaller for smaller atoms). When comparable degrees of restoration are obtained for different locations of the twin element, the probability of twin formation is higher because the twin can form at different stages of crystal growth, corresponding to different atomic surfaces exposed when the twin formation starts. In the opposite case, a higher probability of formation corresponds to the occurrence of a stacking defect, during crystal growth, on a surface corresponding to more restricted, possibly unique, locations of the twin element.

## 4. Case study: the melilite twins   

Melilite is a group of sorosilicate minerals with general formula *X*_2_ *YZ*_2_O_7_ with *X* = Ca, Na, Sr, K in octahedral coordination, *Y* = Mg, Al, Fe, B in tetrahedral coordination and *Z* = Si, Al again in tetrahedral coordination. These minerals crystallize in space groups of type 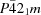 with *X* and *Z* in Wyckoff positions 4*e*, *Y* in Wyckoff position 2*a* and oxygen atoms distributed over three different Wyckoff positions, 2*c*, 4*e* and 8*f*, respectively. We have analysed the structure reported by Bindi & Bonazzi (2005 ▶) for which *a* = 7.826 (1), *c* = 5.004 (1) Å. The atomic coordinates are given in Table 1 ▶, together with an analysis of the quasi-restoration of each orbit. This analysis has been performed with the *PSEUDO* program (Capillas *et al.*, 2011 ▶) at the Bilbao Crystallographic Server (Aroyo *et al.*, 2006 ▶). Given the difference in the dimensions of the cations and the anions, a tolerance of 1 Å for the former and 1.5 Å for the latter has been used to evaluate the pseudo-eigensymmetry.

Two twins in melilite are reported by Deer *et al.* (1997 ▶), with reflections in {001} and {100} as twin operations: both are twins by merohedry so that **L** *_T_* coincides with **L**_ind_. The analysis has to be performed on planes, not on forms, and for this reason in the following the planes (001) and (100) are used; the result is obviously exactly the same if another plane from the same form is used. Since the twins are by merohedry, the intersection group 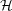 = 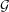 ∩ *t* 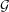 *t* ^−1^ coincides with the group 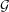 of the individual which is of type 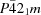 (No. 113). The minimal supergroups containing symmetry operations with the required linear parts are (all symmetry operations are expressed with respect to the standard setting of 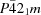):

(1) *P*4/*mbm* (No. 127), with the symmetry operation *m* *x*,*y*,0 for the (001) twin and *b* ¼,*y*,*z* for the (100) twin;

(2) *P*4/*nmm* (No. 129), with *n*(½,½,0) *x*,*y*,0 for the (001) twin and *m* 0,*y*,*z* for the (100) twin;

(3) *P*4_2_/*mnm* (No. 136), with *m* *x*,*y*,¼ for the (001) twin and *n*(0,½,½) ¼,*y*,*z* for the (100) twin;

(4) *P*4_2_/*ncm* (No. 138), with *n*(½,½,0) *x*,*y*,¼ for the (001) twin and *c* 0,*y*,*z* for the (100) twin.

The last two columns in Table 1 ▶ give the respective restoration operations contained in the eigensymmetry of the different orbits.

Both (001) and (100) twins are by merohedry, with the whole lattice restored by the twin operations. The degree of structural restoration is the same for both twins, since the minimal supergroups of 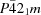 containing a restoration operation for one of the twins also contain one for the other twin. All cation orbits are approximately restored by a reflection located at the origin for the (001) twin and by a *b*-glide reflection shifted ¼ from the origin for the (100) twin, with displacements ranging from 0 (perfect restoration) to 0.6415 Å. On the other hand, all anions are quasi-restored by a reflection shifted ¼ from the origin for the (001) twin and by an *n*-glide reflection shifted ¼ from the origin for the (100) twin, with displacements between 0.0580 and 0.6956 Å. The two further possible restorations for O_3_ correspond to different pseudo-eigensymmetries but the much higher value of *d*_min_ makes their contribution hardly significant.

More recently, a further reflection twin, on (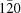), has been reported in melilite by Bindi *et al.* (2003 ▶). The restoration under the action of the twin operation has to be checked in 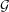 = 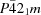 for each orbit *O_i_* [this is easily done by inspecting Table 1 ▶: 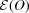 never contains 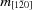] as well as for the union 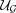 of atoms with similar structural role, *i.e*. *Y* and *Z*, which are both in tetrahedral coordination, and the three types of oxygen atoms (Table 2 ▶). Neither 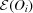 nor 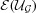 contain 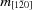 as a proper or pseudo-symmetry which therefore does not restore any orbit or union of orbits under 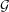. The next step is to check for the restoration of split orbits under 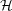.

In a tetragonal lattice, a plane (*hk*0) is exactly perpendicular to the direction with the same indices [*hk*0]; the direction 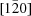 is therefore exactly perpendicular to the twin plane, which can thus also be indicated as 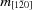. This perpendicularity imposed by the metric of the lattice is known as intrinsic TLS or iTLS (Nespolo & Ferraris, 2006 ▶). Twinning is by reticular polyholohedry, with twin index *n* = 5 (for details, see Nespolo & Ferraris, 2004*b* ▶). The two shortest in-plane directions are [210] and [001] so that the transformation from the basis of the individual to that of the twin, see equation (1), is immediately obtained as follows:
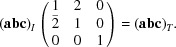
Applying the inverse transformation, the twin plane in the basis of the twin lattice becomes (100) or *m*_[100]_, equation (1′)[Disp-formula fd1a]:
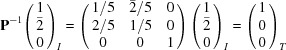
so that the matrix representation **T** of the twin operation *t* in the twin basis is simply:
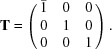
In our case, 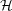 = 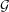 ∩ *t* 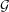 *t* ^−1^ = 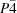, *a* = 17.4995, *c* = 5.0040 Å: in fact, neither the 2-fold axis nor the reflection plane contained in 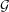 fix the twin lattice, whereas the 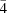 axis does fix it and is common to 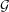 and *t* 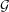 *t* ^−1^.

Let *m*(*O_i_*) be the multiplicity of each orbit *O_i_* in 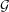, *i* ∈{1, 2, ..6}, and let *n*_i_ be the number of the atoms of the orbit *O_i_* in the unit cell of the twin lattice. Then:
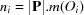
where |**P**| is the determinant of the transformation matrix **P**. The number of atoms *n*_i_, equivalent under 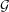, is divided in the twin basis on *s* non-equivalent subsets of atoms under the subgroup 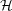: each subset corresponds to a split orbit *O_ij_* indexed by *s* and such that:
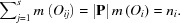
The restoration of a split orbit *O_ij_* is realised when 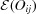 contains a restoration operation with linear part *m*_[100]_*T*__. The extensions of 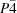 (No. 81) containing such an operation are 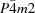 (No. 115), 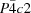 (No. 116), 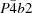 (No. 117) and 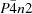 (No. 118); the corresponding restoration operations are *m* 0,*y,z, c* 0,*y,z, b* ¼,*y,z* and *n*(0,½,½) ¼,*y,z*, respectively. To evaluate whether a split orbit under 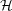 = 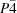 is quasi-restored by the operation in 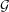, one checks whether one of these four operations maps a split orbit either to itself or to another split orbit of the same type (within the accepted tolerance). This is what is displayed in Tables 3–8 ▶ ▶ ▶ ▶ ▶ ▶. It turns out that the reflection located in the origin gives by far the best restoration results, therefore we will only discuss the restoration by the operation *m* 0,*y*,*z*.

The atoms of type *X* in Wyckoff position 4*e* for 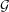 = 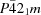 fall under the action of the subgroup 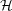 into five split orbits in Wyckoff position 4*h* for 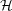 = 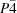, each having four atoms in the unit cell of the twin lattice. The split orbit *X*_1_ is almost perfectly restored (with a deviation of 0.03764 Å), *X*_4_ and *X*_5_ are also quasi-restored with a much larger but still acceptable deviation (0.8617 Å).

The atoms of type *Y* in Wyckoff position 2*a* fall into four split orbits, two of which have four atoms in the twin cell and the other two a single atom. The two split orbits with a single atom in the twin cell are perfectly restored; the split orbit *Y*_4_ is quasi-restored to the split orbit *Z*_3_ with a deviation of 0.6493 Å. This is an admissible replacement, since both the *Y* and the *Z* atoms are in tetrahedral coordination.

The atoms of type *Z* in Wyckoff position 4*e* fall again into five split orbits each having four atoms in the twin cell. Besides the split orbit *Z*_3_ which is interchanged with *Y*_4_, three more split orbits are approximately restored (with deviations between 0.5621 and 0.9793 Å).

The oxygen atoms in Wyckoff position 2*c* fall into two orbits with four atoms in the twin cell and one orbit with two atoms in the twin cell. The split orbit with two atoms is exactly restored, the other two split orbits are only quasi-restored when the threshold for anions is relaxed to 1.5 Å (deviations 1.1740 and 1.3402 Å) and one may doubt whether these are still meaningful for the formation of the twin. The oxygen atoms in Wyckoff position 4*e* fall into five split orbits (each having four atoms in the twin cell). The split orbits O_25_ and O_22_ are approximately restored to themselves (with deviations of 0.5432 and 0.9856 Å), the orbit O_24_ is quasi-restored to the split orbit O_34_ belonging to the oxygen atoms in Wyckoff position 8*f* (with deviation 0.4103 Å) and the remaining two orbits are quasi-restored to different split orbits with deviations between 1 and 1.5 Å. Finally, the oxygen atoms in Wyckoff position 8*f* fall into ten split orbits with four atoms each. Besides the split orbit O_34_ that is interchanged with O_24_, the two orbits O_310_ and O_38_ are quasi-restored to themselves with low deviations (0.1946 and 0.3283 Å). Six more of these split orbits are quasi-restored with higher deviations (between 1 and 1.5 Å).

Table 9 ▶ shows a summary of the above analysis, where we see that the percentage of atoms quasi-restored by the reflection is much better than for the three glide reflections. The fact that 68% of the cations and 37% of the anions are restored within 1 Å is a strong justification for the occurrence of this twin.

**Figure 1:**
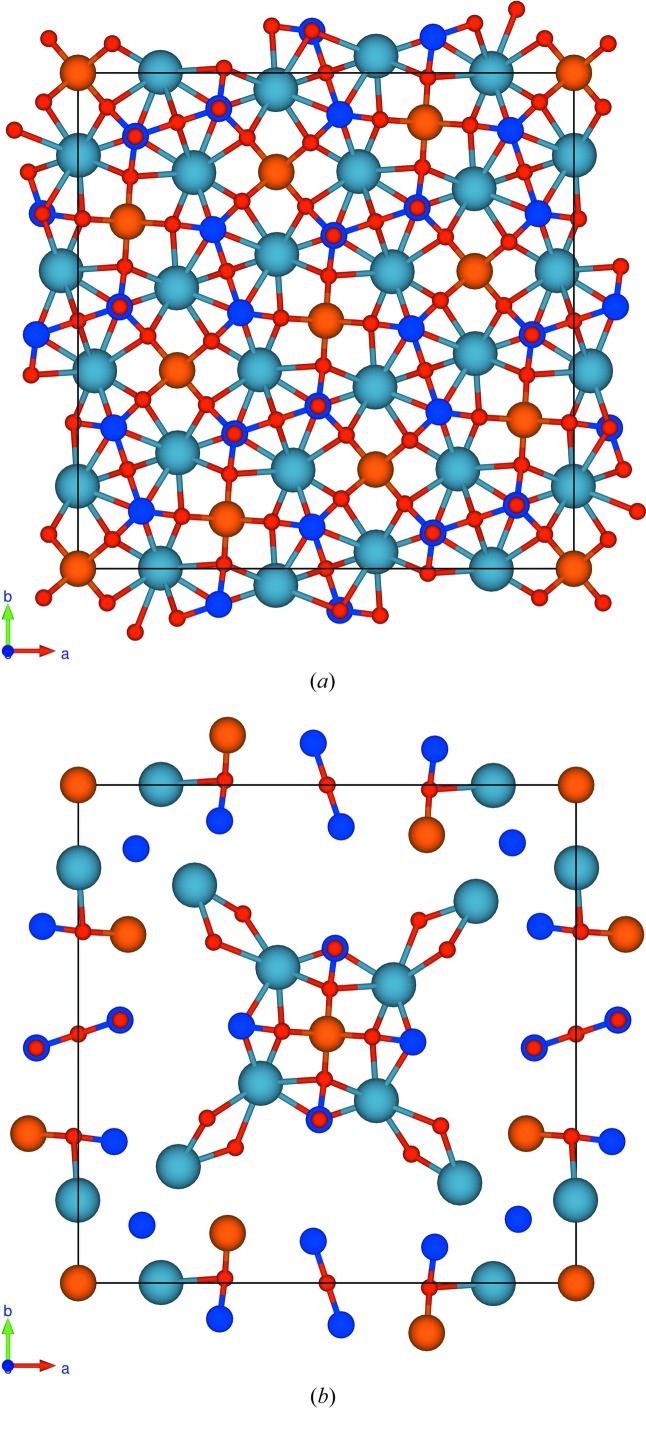
View of the unit cell of the twin lattice of melilite along the *c* axis. The atoms of type *X* (mainly calcium in our example) are coloured light blue, the atoms of type *Y* (mostly magnesium) in orange, the atoms of type *Z* (mainly silicon) dark blue and the oxygen atoms are in red. (*a*) View of all atoms in the cell and (*b*) the quasi-restored atoms.

**Figure 2:**
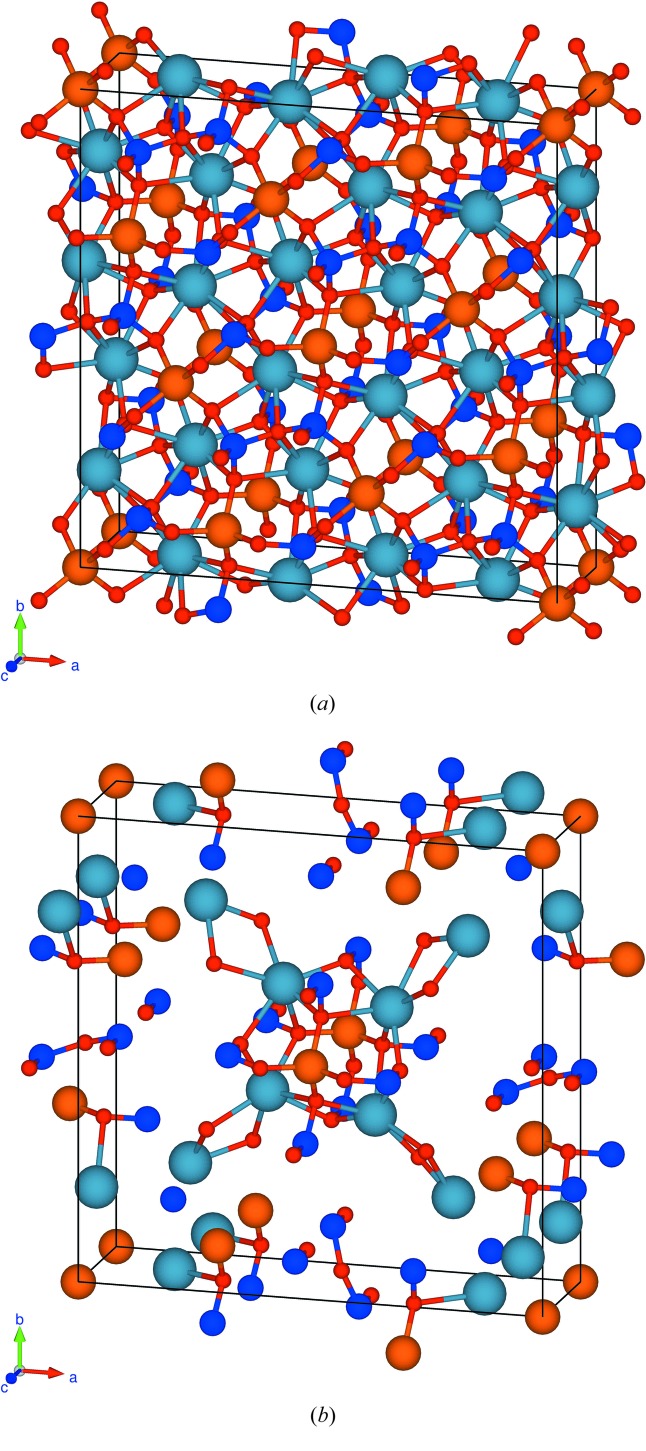
View of the unit cell of the twin lattice of melilite along to the (111) plane: (*a*) all atoms in the cell, (*b*) the quasi-restored atoms.

In Figs. 1 ▶ and 2 ▶ we display views of the twin cell. Figs. 1 ▶(*a*) and 2 ▶(*a*) show all atoms, and Figs. 1 ▶(*b*) and 2 ▶(*b*) the quasi-restored atoms. Fig. 1 ▶ is a view along the *c* axis, *i.e*. the direction of the fourfold rotoinversion axis contained in the subgroup 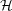; Fig. 2 ▶ is along the normal of the (111) plane.

## 5. Conclusions   

The reticular theory of twinning represents an elegant and general approach for estimating the probability of the occurrence of a twin. However, because it provides a necessary condition only on the lattice level, its application as an *a priori* predictive tool is limited: while a low lattice restoration clearly indicates low probability of formation, a high lattice restoration is indicative, but not conclusive, of a probable occurrence.

The analysis of the eigensymmetry of the crystallographic orbits corresponding to occupied Wyckoff positions is the key for obtaining a quantitative estimation of the structural restoration realised by the twin operation(s) and for obtaining structurally necessary conditions enhancing the reticular conditions for the twin formation. The example of melilite is particularly instructive. The (001) and (100) twins are both twins by merohedry and from the reticular viewpoint both twins should have a high probability of occurrence. As a matter of fact, the structural restoration is also fairly good, although the cations and anions require different locations of the twin element. The 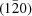 twin, despite a twin index of 5, also leads to a relatively high degree of atomic restoration, which explains the occurrence of this twin.

The approach we have developed in this article opens new perspectives in the study of twins and is currently being applied to other known examples.

## Figures and Tables

**Table 1:** Atomic coordinates of melilite (after Bindi Bonazzi, 2005 ▶) and analysis of the quasi-restoration of each orbit The orbit (pseudo)-eigensymmetry is given as the minimal distance between atoms quasi-restored by the twin operations. This distance coincides with the degree of pseudo-symmetry (_max_) obtained by *PSEUDO* (Capillas *et al.*, 2011 ▶) as the maximal distance between atoms produced by the additional symmetry operations of 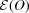. (**P**, **p**) is the matrix-column pair relating the coordinate system of 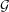 to that of 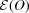. The restoration operations are given with respect to the coordinate system of 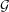.

Site	Wyckoff position	Coordinates	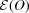	(**P**, **p**)	*d*_min_ ()	Restoration operations for (001) twin	Restoration operations for (100) twin
*X*	4*e*	0.3316(1)	*P*4/*mbm*	(I | 000)	0.0651	*m x,y*,0	*b* ,*y,z*
		0.1684(1)					
		0.5065(2)					
*Y*	2*a*	0	*P*4/*mmm*	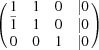	0	*m x,y*,0	*b* ,*y,z *
		0					
		0				*n*(,,0) *x,y*,0	*m* 0,*y,z*
*Z*	4*e*	0.1399(2)	*P*4/*mbm*	(I | 000)	0.6415	*m x,y*,0	*b* ,*y,z*
		0.3601(1)					
		0.9359(3)					
O1	2*c*	0.5	*P4/nmm*	(I | 0)	0	*n*(,,0) *x,y*,0	*m* 0,*y,z*
		0					
		0.1805(9)	*I4/mmm*	(I | 0)	0.6956	*m x,y*, *n*(,,0) *x,y*,0	*n*(0,,) ,*y,z m* 0,*y,z*
O2	4*e*	0.1408(5)	*P4_2_/mnm*	(I | 0)	0.0580	*m x,y*,	n(0,,) ,*y,z*
		0.3592(5)					
		0.2558(9)					
O3	8*f*	0.0795(6)	*P4_2_/mnm*	(I | 0)	0.3643	*m x,y*,	n(0,,) ,*y,z*
		0.1868(5)	*P4_2_/ncm*	(I | )	1.2422	*n*(,,0) *x,y*,	*c* 0,*y,z*
		0.7864(6)	*P4/nmm*	(I | 0)	1.2443	*n*(,,0) *x,y*,0	*m* 0,*y,z*

**Table 2:** Analysis of the eigensymmetry of 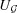, 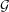 = 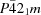

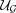	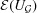	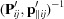	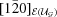
*Y* *Z*	*P*4/*mbm*	(I | 000)	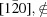
O_1_O_2_	*P*4_2_/*mnm*	(I | 0)	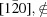
O_1_O_3_	*P*4_2_/*mnm*	(I | 0)	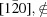
O_2_O_3_	*P*4_2_/*mnm*	(I | 0)	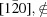
O_1_O_2_O_3_	*P*4_2_/*mnm*	(I | 0)	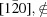

**Table 3:** Analysis of the split orbits *X_j_* stemming from *X* under 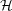 = 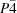 A split orbit *X_j_* is quasi-restored to a split orbit *X_k_* (which may be the same as *X_j_*) by a twin operation if the approximate eigensymmetry 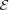 of the union *X_j_* *X_k_* contains (with *d*_min_ within the accepted tolerance) one of 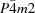, 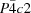, 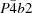 or 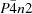, corresponding to the admissible restoration operations *m* 0,*y,z, c* 0,*y,z, b* ,*y,z* and *n*(0,,) ,*y,z*, which are abbreviated as *m, c, b* and *n* in the tables.

Orbit	Coordinates	Wyckoff positions	Restoration operation	Restored to	*d*_min_ ()
*X*_1_	0.99896, 0.16632, 0.5065	4*h*	*m*	*X*_1_	0.0364
*X*_2_	0.19896, 0.56632, 0.5065	4*h*			
*X*_3_	0.39896, 0.96632, 0.5065	4*h*			
*X*_4_	0.79896, 0.76632, 0.5065	4*h*	*m*	*X*_4_	0.8104
*X*_5_	0.59896, 0.36632, 0.5065	4*h*	*m*	*X*_5_	0.8617

**Table 4:** Analysis of the split orbits *Y_j_* stemming from *Y* under 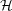 = 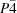 Same conventions as in Table 3 ▶.

Orbit	Coordinates	Wyckoff positions	Restoration operation	Restored to	*d*_min_ ()
*Y*_1_	0, 0, 0	1*a*	*m*	*Y*_1_	0
			*b*	*Y*_2_	0
*Y*_2_	0.5, 0.5, 0	1*c*	*m*	*Y*_2_	0
			*b*	*Y*_1_	0
*Y*_3_	0.2, 0.4, 0	4*h*	*b*	*Z*_3_	0.6493
*Y*_4_	0.9, 0.3, 0	4*h*	*m*	*Z*_3_	0.6493

**Table 5:** Analysis of the split orbits *Z_j_* stemming from *Z* under 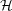 = 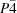 Same conventions as in Table 3 ▶.

Orbit	Coordinates	Wyckoff positions	Restoration operation	Restored to	*d*_min_ ()
*Z*_1_	0.88394, 0.12798, 0.9359	4*h*	*m*	*Z*_1_	0.7061
*Z*_2_	0.08394, 0.52798, 0.9359	4*h*	*m*	*Z*_2_	0.9793
*Z*_3_	0.28394, 0.92798, 0.9359	4*h*	*m*	*Y*_4_	0.6493
			*b*	*Y*_3_	0.6493
*Z*_4_	0.68394, 0.72798, 0.9359	4*h*			
*Z*_5_	0.48394, 0.32798, 0.9359	4*h*	*m*	*Z*_5_	0.5621

**Table 6:** Analysis of the split orbits O_1*j*_ stemming from O_1_ under 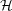 = 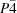 Same conventions as in Table 3 ▶.The restorations with *d*_min_ below 1 are highlighted in bold.

Orbit	Coordinates	Wyckoff positions	Restoration operation	Restored to	*d*_min_ ()
O_11_	0.5, 0, 0.1805	2*g*	*m*	O_11_	**0**
			*n*	O_11_	**0.6956**
O_12_	0.1, 0.2, 0.1805	4*h*	*m*	O_21_	1.3402
			*c*	O_36_	**0.5632**
			*b*	O_34_	1.4183
			*n*	O_35_	1.2773
O_13_	0.3, 0.6, 0.1805	4*h*	*m*	O_35_	1.1740
			*b*	O_36_	**0.2527**
			*n*	O_21_	1.3251

**Table 7:** Analysis of the split orbits O_2*j*_ stemming from O_2_ under 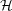 = 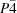 Same conventions as in Table 3 ▶.

Orbit	Coordinates	Wyckoff positions	Restoration operation	Restored to	*d*_min_ ()
O_21_	0.88448, 0.12816, 0.2558	4*h*	*m*	O_12_	1.3402
			*m*	O_31_	1.4748
			*c*	O_21_	**0.3182**
			*n*	O_13_	1.3251
O_22_	0.08448, 0.52816, 0.2558	4*h*	*m*	O_22_	**0.9856**
			*c*	O_32_	1.0051
			*b*	O_37_	1.2569
			*n*	O_22_	1.3950
O_23_	0.28448, 0.92816, 0.2558	4*h*	*m*	O_36_	1.4560
			*c*	O_38_	1.1174
			*b*	O_35_	1.1016
			*n*	O_32_	1.4935
O_24_	0.68448, 0.72816, 0.2558	4*h*	*m*	O_34_	**0.4103**
			*c*	O_24_	1.0825
			*b*	O_39_	1.3750
			*n*	O_36_	1.4279
O_25_	0.48448, 0.32816, 0.2558	4*h*	*m*	O_25_	**0.5432**
			*c*	O_35_	1.1363
			*c*	O_310_	1.4764

**Table 8:** Analysis of the split orbits O_3*j*_ stemming from O_3_ under 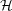 = 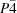 Same conventions as in Table 3 ▶.

Orbit	Coordinates	Wyckoff positions	Restoration operation	Restored to	*d*_min_ ()
O_31_	0.94118, 0.06916, 0.7864	4*h*	*m*	O_21_	1.4748
			*c*	O_31_	**0.4452**
			*b*	O_310_	1.0048
			*n*	O_310_	1.4670
O_32_	0.14118, 0.46916, 0.7864	4*h*	*m*	O_32_	1.0794
			*c*	O_22_	1.0051
			*b*	O_38_	1.3284
			*n*	O_23_	1.4935
O_33_	0.34118, 0.86916, 0.7864	4*h*	*m*	O_39_	1.2177
			*c*	O_37_	1.1345
			*n*	O_33_	**0.7824**
O_34_	0.74118, 0.66916, 0.7864	4*h*	*m*	O_24_	**0.4103**
			*c*	O_39_	1.3239
			*b*	O_12_	1.4183
O_35_	0.54118, 0.26916, 0.7864	4*h*	*m*	O_13_	1.1740
			*m*	O_35_	1.4413
			*c*	O_25_	1.1363
			*b*	O_23_	1.1016
			*n*	O_12_	1.2773
			*n*	O_38_	1.4677
O_36_	0.89444, 0.20938, 0.7864	4*h*	*m*	O_23_	1.4560
			*c*	O_12_	**0.5632**
			*b*	O_13_	**0.2527**
			*n*	O_24_	1.4279
O_37_	0.09444, 0.60938, 0.7864	4*h*	*c*	O_33_	1.1345
			*b*	O_22_	1.2569
			*n*	O_37_	**0.5190**
O_38_	0.29444, 0.00938, 0.7864	4*h*	*m*	O_38_	**0.3283**
			*c*	O_23_	1.1174
			*b*	O_32_	1.3284
			*n*	O_35_	1.4677
O_39_	0.69444, 0.80938, 0.7864	4*h*	*m*	O_33_	1.2177
			*c*	O_34_	1.3239
			*b*	O_24_	1.3750
			*n*	O_39_	**0.3764**
O_310_	0.49444, 0.40938, 0.7864	4*h*	*m*	O_310_	**0.1946**
			*c*	O_25_	1.4764
			*b*	O_31_	1.0048
			*n*	O_31_	1.4670

**Table 9:** Summary of the percentage of atomic quasi-restoration by the 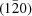 twin plane in melilite for the admissible restoration operations (expressed in the basis of the twin) The values in parentheses are obtained by also taking into account the oxygen atoms restored with a degree of approximation between 1 and 1.5. In the unit cell of the twin lattice, there are 20 cations of type *X*, 10 cations of type *Y*, 20 cations of type *Z* and 70 oxygen atoms, thus in total 120 atoms.

Restoration operation	%*X*	%*Y*	%*Z*	% cations	%O	% all atoms
*m* 0,*y,z*	60	60	80	68	37 (94)	50 (83)
*c* 0*,y,z*	0	0	0	0	23 (91)	13 (53)
*b* ,*y,z*	0	60	20	20	11 (80)	15 (55)
*n*(0,,) ,*y,z*	0	0	0	0	20 (89)	12 (52)

## Footnotes

1 For manifold twins (*i.e.* twins in which the twin operation is higher than twofold), a zero-obliquity TLQS may occur. In this case, a different parameter is necessary to measure the deviation from the exact restoration of lattice nodes, like the twin misfit introduced by Nespolo & Ferraris (2007 ▶).

## Appendix A

**Theorem**. Assume^2^ that *t* is the twin operation such that *t* ^2^ is an element of . Let *O_ij_* be a split orbit under the intersection group  =  **∩** *t*  *t* ^−1^ and let **x** be the position of an atom in *O_ij_*. Let **x**′ be the position of the atom in the structure closest to the mapped position *t*(**x**) of **x** under the twin operation, thus *d*_min_ = ∥*t*(**x**) − **x**′∥. Then the value of *d*_min_ is the same for every atom in *O_ij_*, *i.e.* the distance of the image of any atom in *O_ij_* under *t* to the closest atom position in the structure is always *d*_min_.

Moreover, if the position **x**′ belongs to the split orbit *O_i′j′_*, then the closest atoms to the mapped split orbit *t*(*O_ij_*) all belong to *O_i′j′_*. In particular, if one atom of *O_ij_* is exactly restored to an atom in *O_i′j′_*, then the full split orbit *O_ij_* is mapped to the full split orbit *O_i′j′_* under the twin operation.

**Proof**: Let **x** be the position of an atom in *O_ij_*, let **x**′ be the position of the atom in the structure closest to *t*(**x**) and let the split orbit to which **x**′ belongs be *O_i′j′_*. If **y** is the position of another atom in *O_ij_*, then there is a symmetry operation *h* in  mapping **x** to **y**. Since *t* is a twofold twin operation, one has *tht* ^−1^ ∈ *t*  *t* ^−1^ ∩ *t* ^2^  *t* ^−2^ = *t*  *t* ^−1^ ∩  =  and hence *tht* ^−1^ = *h*′ ∈ . This means that *th* = *h*′*t* and thus mapping **y** = *h*(**x**) by the twin operation *t* gives *t*(**y**) = *th*(**x**) = *h*′*t*(**x**). If one defines **y**′ = *h*′(**x**′), then from the fact that *h*′ is an isometry and thus preserves distances, it follows that ∥*t*(**y**) − **y**′∥ = ∥*h*′*t*(**x**) − *h*′(**x**′)∥ = ∥*h*′[*t*(**x**) − **x**′]∥ = ∥*t*(**x**) − **x**′∥ = *d*_min_. Since *h*′ is an element of , it follows that *O_i′j′_* contains an atom with distance *d*_min_ to *y*. The same argument applied with the roles of *O_ij_* and *O_i′j′_* interchanged now shows that the structure can not contain an atom closer to *t*(**y**) than **y**′, because that would result in an atom with distance less than *d*_min_ to *t*(**x**).

**Remark**: The above proof is easily generalized to the case of a *k*-fold twin. In this case, the intersection subgroup has to be chosen as  =  **∩** *t*  *t* ^−1^ **∩** *t* ^2^  *t* ^−2^ **∩** … **∩** *t* ^*k* − 1^  *t* ^−(*k* − 1)^. Then the crucial argument in the proof that *tht* ^−1^ = *h*′ ∈  remains valid.
